# A Shot in the Dark: Unprecedented Complete Radiologic Response of Metastatic Prostate Cancer to Single-Dose Androgen Deprivation Therapy (ADT)

**DOI:** 10.7759/cureus.104755

**Published:** 2026-03-06

**Authors:** Rohit S Deshpande, Puneet Ahluwalia

**Affiliations:** 1 Robotic Surgery and Uro-Oncology, Medanta - The Medicity, Gurugram, Gurugram, IND

**Keywords:** androgen deprivation therapy, metastatic, prostate cancer, psa, psma-pet-ct

## Abstract

Androgen deprivation therapy (ADT) is a well-established treatment strategy for metastatic prostate cancer, aimed at reducing tumor burden and alleviating symptoms. Although ADT is a cornerstone in managing prostate cancer, complete radiological regression of metastatic disease, with a single dose of ADT, has not been previously reported. We present an exceptional case of a patient with metastatic prostate cancer who achieved a complete radiological response to ADT, highlighting an extraordinary therapeutic outcome.

## Introduction

It is common knowledge that androgen sensitivity is one of the hallmark features of prostate cancer, as proven by Dr. Charles Huggins in 1941, when he and Clarence Hodges suggested that “significant improvement often occurs in the clinical condition of patients with far advanced cancer of the prostate after they have been subjected to castration. Conversely, the symptoms are aggravated when androgens are injected” [1]. However, complete radiological regression of metastatic disease has never been reported so far in medical literature.

We present one such case wherein an elderly gentleman with metastatic prostate cancer was started on androgen-deprivation therapy (ADT), and was found to have a complete radiological response on subsequent imaging.

## Case presentation

**Figure 1:**
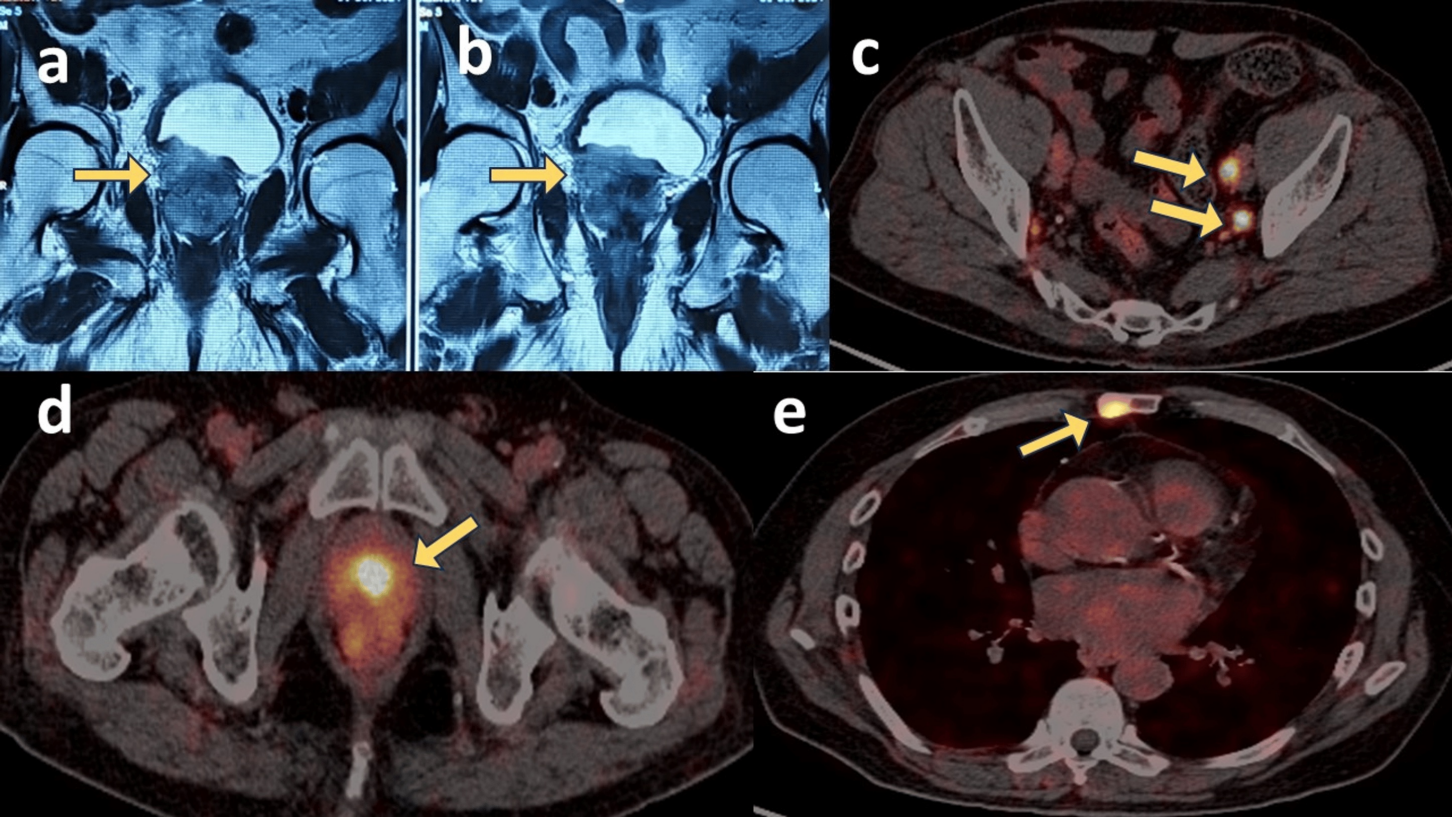
MpMRI and PSMA PET-CT appearances of the prostatic primary and metastatic sites a: There was diffuse nodularity of the central and transitional zones of the prostate (88 cc in volume) with heterogeneous parenchyma. b: The right-side base of the peripheral zone was moderately bulky and showed a well-defined, nodular lesion in the median and para-medial locations. The lesion measured approximately 3.7 x 3 cm, was isointense on T1W (T1-weighted) images, hypointense on T2W (T2-weighted) sequences, with restricted diffusion, hyperintense on DWI (diffusion-weighted imaging), and darkening on ADC (apparent diffusion coefficient). The lesion showed intense enhancement on T1WFS (T1-weighted fat-saturated) post-gadolinium images. The prostatic capsule was breached, and neurovascular bundles were infiltrated. The right seminal vesicle and levator ani were bulky, with loss of planes suggestive of infiltration. There was a contiguous infiltration of the bladder base and right lateral wall. c: Multiple PSMA-avid pelvic lymph nodes on PSMA PET-CT scan. d: Intense PSMA-avidity of the prostatic primary malignancy. e: PSMA-avid sternal lesion (metastatic site). MpMRI: multiparametric magnetic resonance imaging; PSMA PET-CT: prostate-specific membrane antigen positron-emission tomography-computed tomography

An elderly Asian gentleman presented to the outpatient department with storage-type lower urinary tract symptoms. Upon evaluation, he was found to have an enlarged prostate on ultrasound, with a serum prostate-specific antigen (PSA) level of 43 ng/ml. Multiparametric magnetic resonance imaging (MpMRI) revealed a significantly enlarged prostate (88 gm) with multiple prostate imaging reporting and data system (PIRADS)-5 lesions (Figure 1) infiltrating into the right bladder wall, right seminal vesicle, and neurovascular bundles, along with right obturator lymphadenopathy. Prostate-specific membrane antigen positron-emission tomography computed tomography.

Prostate-specific membrane antigen positron-emission tomography-computed tomography (PSMA PET-CT) done for metastatic workup revealed the prostatic primary with multiple enlarged pelvic lymph nodes and sternal metastasis (confirmed by a radiologist) (Figure 1). Trans-rectal ultrasound (TRUS)-guided systematic 12-core biopsy revealed 12/12 cores positive for acinar adenocarcinoma with a Gleason score of 3+4=7 and International Society of Uro-Pathology (ISUP) grade group II.

Subsequently, as part of ADT for metastatic disease, hormonal therapy was started with a single injection of luteinizing hormone-releasing hormone (LHRH) agonist (leuprolide acetate 22.5 mg).

**Figure 2:**
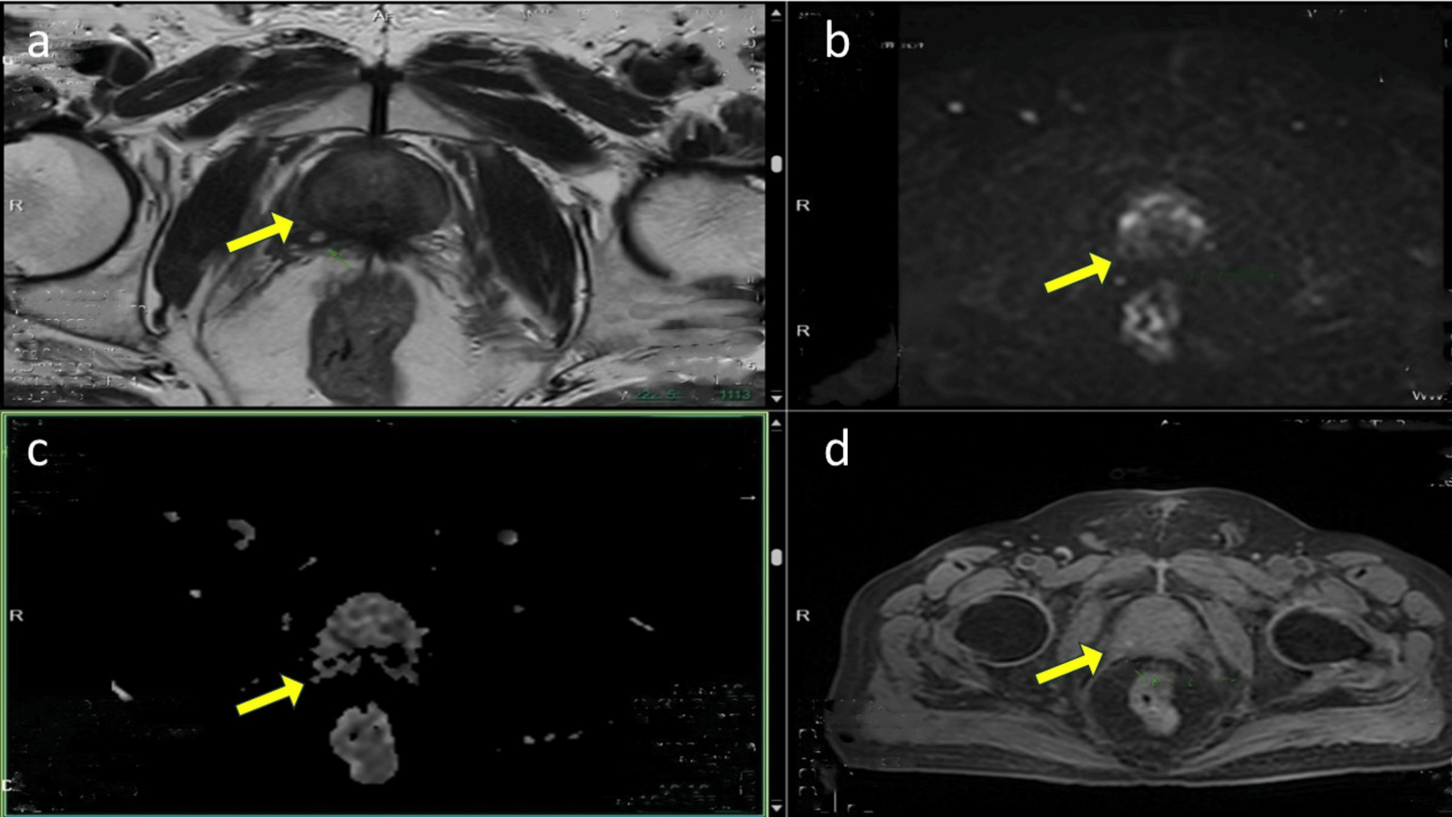
Post-ADT MpMRI prostate a: Ill-defined T2 hypointensity in the right posterolateral aspect of the peripheral zone in mid-body extending to the apex, representing an area of post-treatment scarring/desmoplastic reaction. b and c: The prostatic lesion showed no evidence of diffusion restriction/ADC hypointensity or differential focal enhancement. Fat planes with adjacent levator ani muscles appeared well-maintained. No evidence of enlarged pelvic lymph nodes. d: The periprostatic-perivesical fascia were unremarkable. There was no free fluid in the pelvis. The pelvic side walls were free, and the bony pelvis was unremarkable. Features were suggestive of a PRECISE score of 2. ADT: androgen deprivation therapy; MpMRI: multiparametric magnetic resonance imaging; ADC: apparent diffusion coefficient; PRECISE: Prostate Cancer Radiological Estimation of Change in Sequential Evaluation

**Figure 3:**
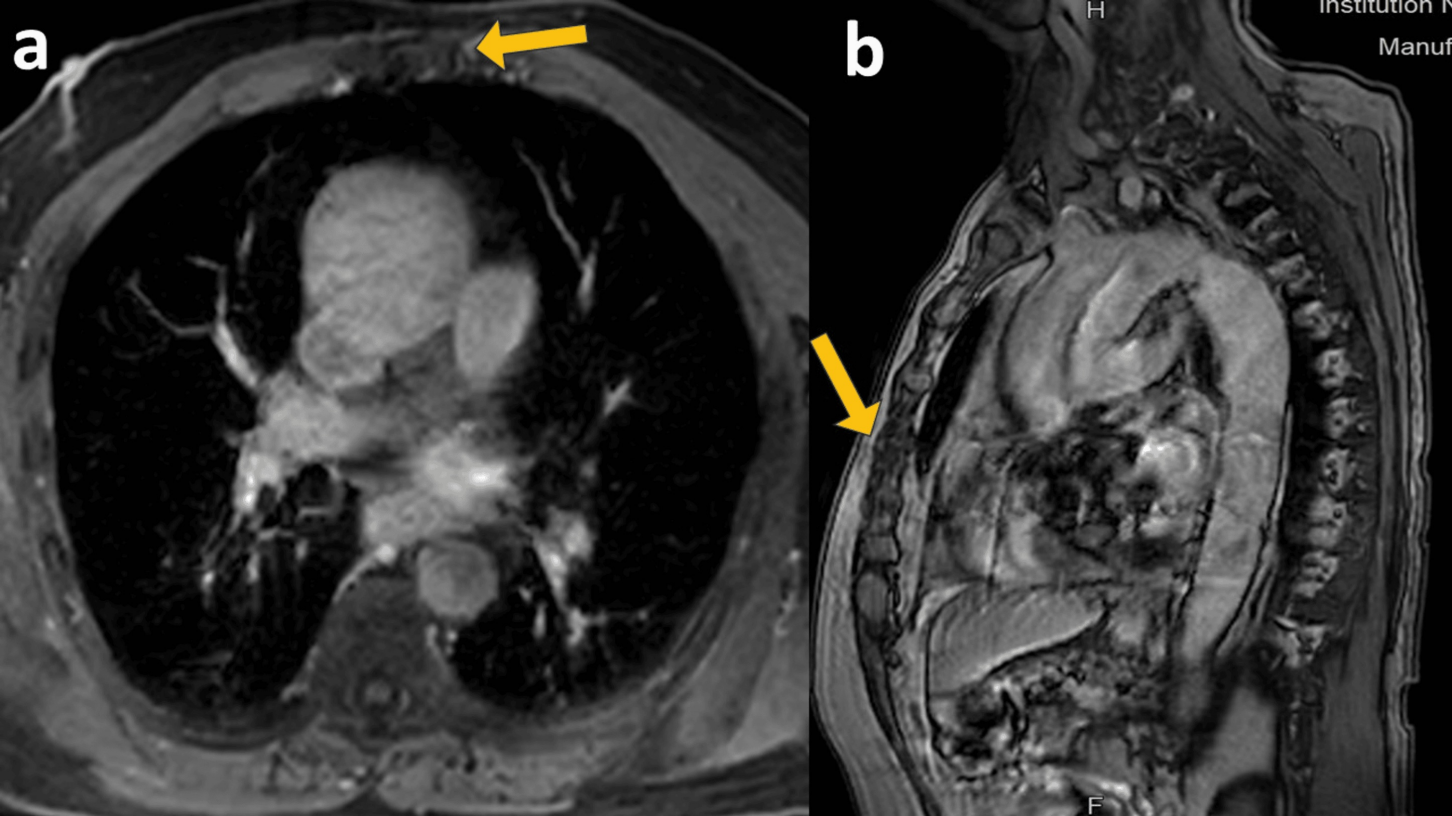
Post-ADT screening MRI of sternal metastatic lesion Screening MRI of the sternal lesion revealed no diffusion restriction, i.e., resolution of sternal metastases. ADT: androgen deprivation therapy

At the follow-up visit (scheduled for three months after presentation), the patient reported complete resolution of lower urinary tract symptoms. Repeat PSA level was 0.11 ng/ml. Repeat imaging (Figure 2) consisting of MpMRI (done to assess disease response to hormonal therapy) with screening of the sternal lesion (Figure 3) revealed a 27-gm prostate with no evidence of disease, a PRECISE (Prostate Cancer Radiological Estimation of Change in Sequential Evaluation) score of 2 (indicating radiological regression) [2], and no evidence of diffusion restriction in the sternal lesion.

## Discussion

There have been few studies determining the radiologic changes in prostate cancer after ADT. According to a study done by Barrett et al., all parameters of dynamic contrast-enhanced imaging on MRI, measured in regions of interest at the sites of tumour, significantly decreased after the administration of three months of ADT. They also found that the median PSA level drastically reduced from a pre-treatment value of 30 ng/mL to 1.5 ng/mL in the post-treatment scenario. Also, the median prostatic volume underwent reduction from 47.6 cm^3^ (pre-treatment value) to 24.9 cm^3^ (post-treatment value); p < 0.001. According to their findings, the MRI parameter of DWI may bear more accuracy in identifying the presence of prostatic tumor cell death (as opposed to residual prostatic tumor). Dynamic contrast-enhancement (DCE) imaging, when used as a surrogate measure of neovascularization or angiogenesis, may greatly facilitate a demonstration of ADT resistance [3].

According to Kojima et al., the maximal effect of ADT occurs in the time period between three and four months [4]. It has been generally reported that the entire volume of the prostate decreases by 10-52% [5-7]. A study by Nemoto et al. investigated how antiandrogen medication influenced diffusion-weighted images that were used to visualize prostate cancer. The time-course changes in the results from DWI (in comparison to those from T2-weighted images) were studied [8]. TRUS-guided imaging and PSA kinetics were examined after patients had received antiandrogen therapy for six months [8]. They arrived at the conclusion that DWI helped understand the clinical significance of PSA kinetics in patients having low PSA biochemical recurrence, while still receiving antiandrogen therapy.

## Conclusions

This case report describes an exceptional and hitherto-unreported full radiological remission of metastatic prostate cancer following a single dosage of androgen-deprivation therapy (ADT). Prostate cancer's androgen sensitivity has long been known, but current literature has never seen such an overwhelming radiologic response, which was demonstrated by near-complete normalization of prostate size and resolution of metastatic lesions. PSMA PET-CT and multiparametric MRI were essential for both the initial diagnosis and the evaluation of the therapy response. The only limitation of this case report was the limited duration of follow-up. This instance highlights the significant influence of ADT and the value of imaging in analyzing the effectiveness of treatment. It also resurrects research questions about biomarkers of severe responders, the best imaging follow-up, and possible ramifications for therapeutic customization in metastatic prostate cancer.
